# Effect of subcutaneous fat on long-term survival after surgery for stage I-III breast cancer as determined by computed tomography

**DOI:** 10.3389/fonc.2025.1506629

**Published:** 2025-03-17

**Authors:** Yi-Wen Mao, Hong-Dou Zeng, Ye Fang, Xin-Yao Wu, Ming-Hao Zhang, Cheng-Da Hu, Ya-Xin Zhao

**Affiliations:** ^1^ Department of Breast Surgery, The Second Affiliated Hospital and Yuying Children’s Hospital of Wenzhou Medical University, Wenzhou, China; ^2^ Department of Gastroenterology, The Second Affiliated Hospital and Yuying Children’s Hospital of Wenzhou Medical University, Wenzhou, China

**Keywords:** body composition, obesity, subcutaneous fat tissue, breast cancer, prognosis, nutrition

## Abstract

**Introduction:**

Monitoring body composition through Computed Tomography (CT) scans, including muscle and adipose tissue, plays a key role in the prognosis of various cancers. However, abdominal CT is impractical for patients with breast cancer (BC), making chest CT an essential tool for postoperative surveillance. This study aims to evaluate the effect of subcutaneous fat tissue at the 11th thoracic vertebral plane on the postoperative prognosis of BC patients by analyzing chest CT images, providing evidence for postoperative nutritional and rehabilitation guidance.

**Methods:**

We conducted a retrospective analysis of the medical records of 188 BC patients treated and discharged from the Second Affiliated Hospital of Wenzhou Medical University between January 1, 2013, and December 31, 2013. The subcutaneous fat area (SFA) at the 11th thoracic vertebra (T11) was measured using chest CT images, and the subcutaneous fat index (SFI, area/height^2^) was calculated. Using multivariate Cox proportional hazards models and propensity score matching (PSM), the relationships between the SFI and overall survival (OS), as well as recurrence-free survival (RFS), were assessed. Additionally, Kaplan-Meier survival curves were applied to compare prognostic differences between the groups.

**Results:**

The median follow-up duration was 128 months (range: 27-188 months). Of the 188 patients included in the study, the optimal cutoff value for the SFI was determined to be 49.31 cm²/m². Multivariate analysis indicated that SFI was an independent prognostic factor for both OS (HR 2.50, 95% CI 1.07-5.83, *P* = 0.034) and RFS (HR 2.04, 95% CI 1.10-3.78, *P* = 0.024). After PSM, Kaplan-Meier survival curve analysis revealed significant differences in both RFS and OS between the two groups (P = 0.025 and P = 0.018, respectively). All the results showed that the prognosis of BC with more subcutaneous fat was poor.

**Discussion:**

The findings demonstrated that the SFI at T11 was negatively correlated with patient survival. This offers a new perspective on personalized management for BC patients, suggesting that future research should validate these results and investigate combining imaging assessments with lifestyle interventions, such as exercise, nutrition, and diet, to optimize patient outcomes.

## Graphical Abstract

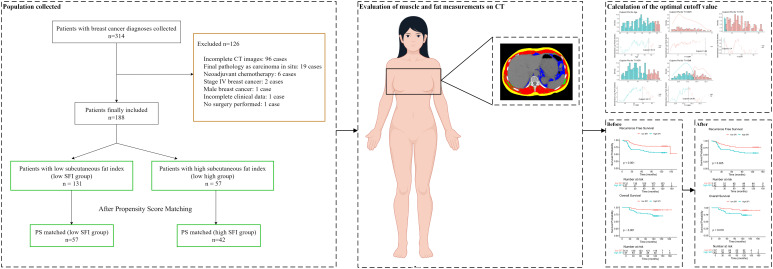

## 1 Introduction

Breast cancer (BC) has become one of the most prevalent cancers among women globally (1, 2). According to the latest cancer statistics projections (3), the incidence of BC is increasing by approximately 1% annually. By 2025, BC is expected to account for 32% of new cancer cases, ranking first, and 14% of cancer-related deaths, ranking second. Despite continuous advancements in treatment, some BC patients continue to experience poor prognoses, including disease recurrence or metastasis (4, 5). To improve these outcomes and enable early prediction and effective intervention, the identification of new biomarkers is essential. In the management and treatment of BC, body composition, including muscle and adipose tissue, is increasingly recognized as a critical predictor of long-term prognosis (6–9). For instance, a lower pectoralis major index at the fourth thoracic vertebra (T4) has been linked to poorer distant metastasis-free survival (DMFS) and overall survival (OS) in BC patients (10). However, such indicators are often overlooked in clinical practice, and the specific role of subcutaneous fat remains unclear (11).

Obesity is a well-established pathogenic and prognostic factor in BC and is significantly associated with poorer long-term survival outcomes (12–16). Previous research has shown that obesity increases the risk of BC recurrence and mortality by 35% to 40%. An analysis of 221 datasets revealed that for every 5 kg/m² increase in body mass index (BMI) in postmenopausal women, the risk ratio for developing BC increased by 1.12% (12). While BMI, which measures body fatness based on weight and height, is commonly used to assess obesity, it is not a comprehensive measure of body composition. BMI does not differentiate between fat and muscle, nor does it reflect the distribution of adipose tissue (17–20). Therefore, relying solely on BMI for clinical evaluation may obscure important conditions, such as sarcopenia and hyperlipemia, which could mask each other (21). These findings emphasize the need for more precise assessments of body fat’s impact on BC prognosis. Visceral fat and subcutaneous fat, two distinct types of body fat, differ significantly in their pathology and physiology, leading to significantly different biological effects (22, 23). Visceral fat is closely associated with metabolic syndrome and cardiovascular disease (24, 25), while subcutaneous fat plays a crucial role in fat distribution, particularly in premenopausal women (26). Recent studies have increasingly focused on the role of subcutaneous fat in cancer patients, yet the specific influence of subcutaneous fat on BC prognosis remains unclear.

With the advancement of imaging techniques like computed tomography (CT), it is now possible to obtain cross-sectional images of any part of the body and assess body composition (11, 27, 28). This progress enables large-scale body composition assessments, making them a potential part of routine clinical care (29). Most studies that provide detailed assessments of body composition through CT focus on the cross-sectional area of fat and muscle at the third lumbar spine (L3) level. For instance, Caan et al. found that in patients with non-metastatic BC, overall mortality was higher in those with sarcopenia and a high total adipose tissue (TAT) index measured at L3 (6). Similarly, Deluche et al. reported that greater intermuscular fat tissue and lower skeletal muscle areas, as measured by CT at the L3 level, were associated with poorer prognosis in early BC (11). Although body composition analysis at the L3 plane may predict OS in BC patients who undergo routine abdominal imaging, UK guidelines recommend that CT scans be performed in BC patients only if there is a significant risk of metastasis (30). Consequently, obtaining L3-level CT images is not always feasible for BC patients. Chest CT, used for preoperative preparation and postoperative review in BC cases, plays a more practical role in clinical assessment. In light of this, the eleventh thoracic vertebra (T11) plane was selected as an alternative point for body composition analysis. T11, located within the thoracic cavity, is routinely included in chest CT scans for BC patients and captures key muscle groups such as the intercostal muscles and erector spinae, while also providing reliable adipose tissue data. Additionally, a deep learning radiomics (DLR) approach has demonstrated that muscle and fat measurements at the T4 and T11 planes significantly impact distant metastasis and mortality in BC (31, 32).

Although previous studies have examined the impact of body composition on BC prognosis, most have focused on BMI and sarcopenia, overlooking the specific role of subcutaneous fat. Additionally, most of these studies have assessed body composition at the L3 plane. However, BC patients typically do not undergo CT scans of the L3 region, which limits the broader applicability of the findings (30). The omission of subcutaneous fat’s potential impact on prognosis may result in the exclusion of important predictive information during clinical evaluations. Therefore, the aim of this study was to investigate the relationship between the T11 subcutaneous fat index (T11SFI) and prognosis, including OS and recurrence-free survival (RFS), in patients with invasive BC post-surgery.

This study evaluated the influence of T11 subcutaneous fat on BC prognosis using propensity score matching (PSM). Through a retrospective analysis, subcutaneous fat at the T11 plane was quantified using CT images, and its association with prognostic indicators such as RFS and OS was explored. This study addresses the limitations of previous research by offering a more precise method for evaluating subcutaneous fat and aims to provide a more accurate prognostic tool for clinical practice.

## 2 Materials and methods

**Figure 1:**
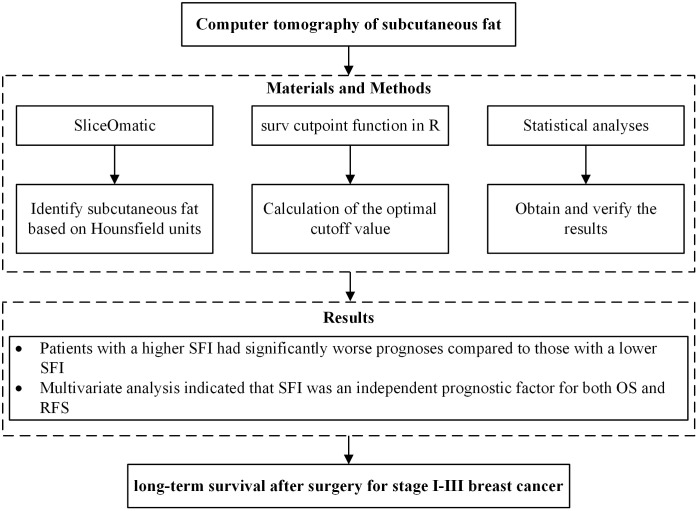
Study flow chart. OS, overall survival; RFS, recurrence-free survival.

We created a flow chart to visualize our research process, as shown in **Figure 1**. Using SliceOmatic software, we identified subcutaneous fat based on Hounsfield units, and employed the surv-cutpoint function in R to determine the optimal cutoff value. We then conducted statistical analyses, including multivariate Cox regression, propensity score matching, and Kaplan-Meier survival curves, to ensure the robustness of the results. These methods allowed us to assess the independent impact of the SFI on OS and RFS.

### 2.1 Study population

We retrieved the medical records of 314 BC patients discharged between January 1, 2013, and December 31, 2013, from the electronic medical records system of the Second Affiliated Hospital of Wenzhou Medical University. The inclusion criteria required patients to have been pathologically diagnosed with invasive BC after surgery and to have undergone a chest CT examination within two years post-surgery, with the CT images including the 11th thoracic vertebra. We excluded patients with incomplete CT imaging (96 cases), carcinoma *in situ* (19 cases), those who received neoadjuvant chemotherapy (6 cases), stage IV BC (2 cases), male BC (1 case), those with incomplete clinical data (1 case), and patients who had not undergone surgery (1 case).

The following patient data were extracted from the electronic medical record system: age at diagnosis, height, weight, BMI, body surface area (BSA), nutritional risk screening 2002 (NRS2002), BC pathological stage, pathological features, treatment, hemoglobin levels, total protein, albumin, comorbid hypertension, comorbid diabetes mellitus, date of last visit, and death records. The collected data were retrospectively analyzed for this study. The study was reviewed and approved by the Ethics Review Committee of the Second Affiliated Hospital of Wenzhou Medical University, and the requirement for written informed consent was waived (Approval NO. 2024-K-014-01).

### 2.2 Evaluation of muscle and fat measurements on CT

**Figure 2:**
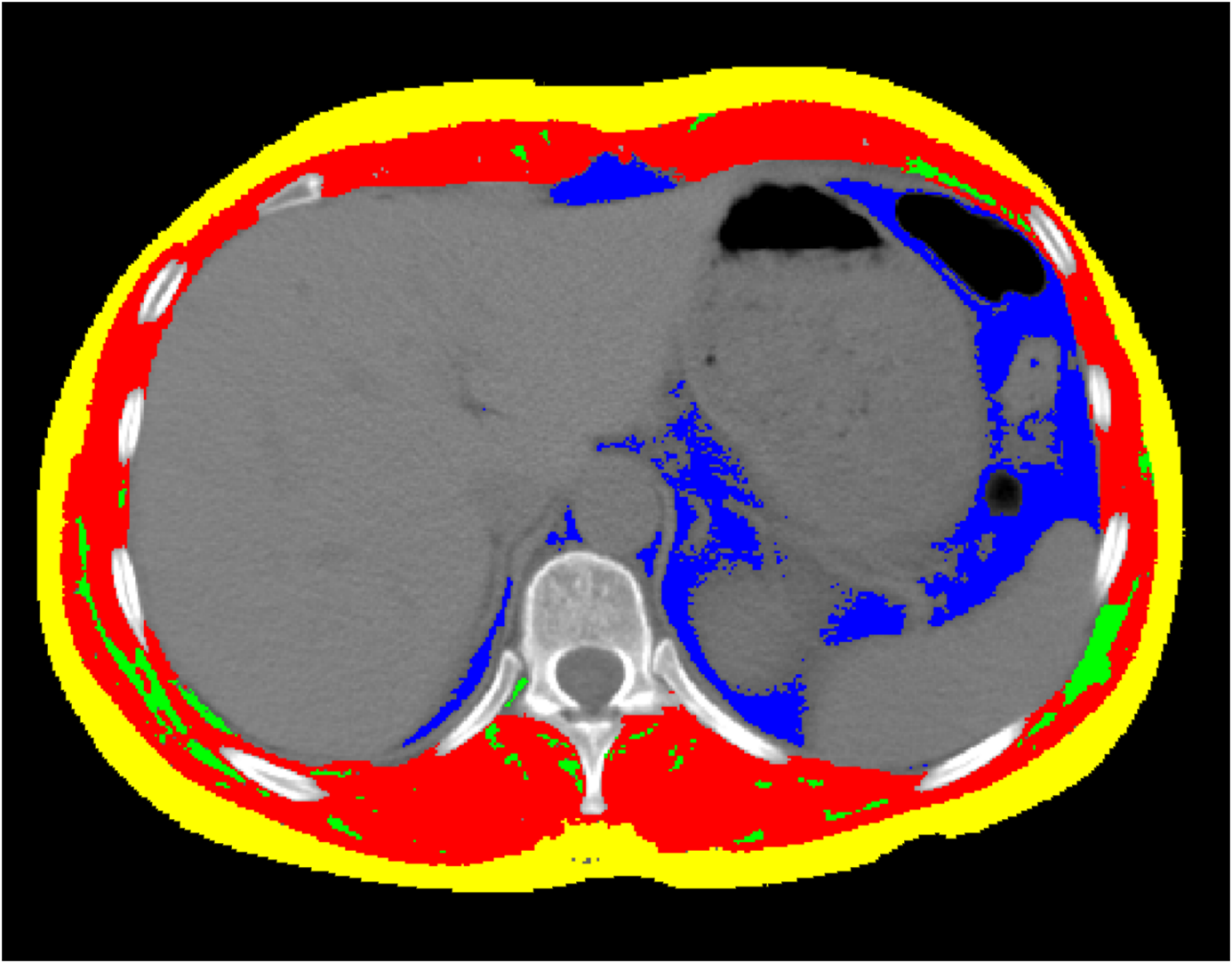
The CT image of the 11th thoracic vertebral plane. Images of cross-sectional area of skeletal muscle (Red colored area) with the Hounsfield unit (HU) from − 29 to + 150, and of intermuscular fat tissue (Green colored area) from − 190 to − 30, and of subcutaneous fat tissue (Yellow colored area) from − 190 to − 30, and of visceral fat tissue (Blue colored area) from − 150 to − 50, using the SliceOmatic Software version 5.0 (Tomovision, Montreal, QC, Canada). The colored regions are the regions of interest.

A centrally trained medical professional used SliceOmatic Software version 5.0 (Tomovision, Montreal, QC, Canada) to identify body tissues based on Hounsfield units (HU) (6, 33). Transverse CT sections of the T11 were determined using bony markers, including the 11th rib, the second vertebra above the 1st lumbar vertebra, and the 10th vertebra below the 1st thoracic vertebra. The uppermost image showing a complete circular foramen was selected for analysis. After selecting the appropriate T11 vertebral body level in the chest CT, muscle and adipose tissues were differentiated and labeled according to their CT values (HU) and anatomical position (**Figure 2**). Tissues with HU values ranging from -29 to +150 were identified as muscle, including all skeletal muscles at this level, such as the intercostal muscles, external oblique abdominal muscles, rectus abdominis, transverse abdominis, erector spinae, and latissimus dorsi muscles. Subcutaneous and intermuscular fat tissues were defined by HU values between -190 and -30, while visceral fat tissue was identified with HU values between -150 and -50. The software automatically calculated the skeletal muscle area (SMA), visceral fat area (VFA), subcutaneous fat area (SFA), and intermuscular fat area (IMFA). To account for height, the obtained area was divided by the square of height (in meters), yielding indices such as the skeletal muscle index (SMI) = SMA (cm²)/height² (m²), as well as the visceral fat index (VFI), SFI, and intermuscular fat index (IMFI).

### 2.3 Calculation of the optimal cutoff value

**Figure 3:**
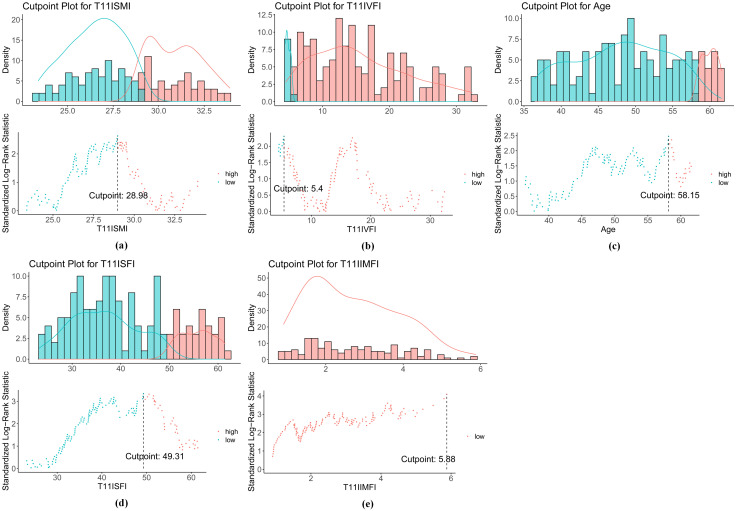
Optimal cutoff values for **(a)** SMI, **(b)** VFI, **(c)** age, **(d)** SFI, and **(e)** IMFI. SMI, skeletal muscle index; VFI, visceral fat index; SFI, subcutaneous fat index; IMFI, intermuscular fat index.

In clinical practice, there are no established thresholds for SMI, VFI, SFI, and IMFI, as these variables are typically treated as continuous. To establish appropriate groupings, we used a survival analysis method to determine the optimal cutoff values based on the maximization of statistical differences. This was achieved by applying the surv_cutpoint function in R, using survival data (recurrence time and time of death). The results indicated that the optimal cutoffs for SMI (**Figure 3a**), VFI (**Figure 3b**), SFI (**Figure 3d**), and IMFI (**Figure 3e**) were 28.98, 5.40, 49.31, and 5.88 cm^2^/m^2^, respectively. These cutoffs allowed us to categorize each variable into high and low groups, enabling further analysis of their relationships with patient outcomes. We also calculated the optimal cutoff value for age, which was 58.15 years (**Figure 3c**). Since age is typically considered as an integer, we selected 58 years as the cutoff point for age grouping. Additionally, overweight patients were defined as having a BMI ≥ 23 kg/m² (34, 35).

### 2.4 Statistical analyses

The primary endpoints of this study were OS and RFS. Baseline characteristic differences among SFI groups were compared using T-tests, Chi-square tests, and the Wilcoxon rank sum test as appropriate. Continuous variables are presented as means with standard deviations, while categorical variables are reported as frequencies and percentages. Kaplan–Meier survival curves were used to assess the prognosis of the two groups, and differences between the survival curves were evaluated using the log-rank (Mantel-Cox) test. To determine the hazard ratios (HR) for RFS and OS, univariate and multivariate analyses were performed using the Cox proportional hazards regression model. Variables identified in the univariate analysis were included in the multivariate Cox regression model through a backward stepwise selection process to assess the prognostic effect of each factor on the study’s two primary endpoints. Propensity score matching (PSM) was employed, a common method in retrospective research, to adjust for various baseline parameters and simulate the outcomes of a hypothetical randomized study. To enhance the credibility and validity of the results, PSM was conducted using a 1:2 nearest-neighbor matching scheme with a caliper width of 0.1. This approach minimizes the impact of potential confounding factors and reduces selection bias between the high and low SFI groups. Statistical significance was defined as *P* < 0.05, with a 95% confidence interval (CI). Statistical analyses were conducted using IBM SPSS Version 23.0, and R 4.3.3 was used to generate graphical representations.

## 3 Results

### 3.1 Patient characteristics

We analyzed the medical records of 314 BC patients and selected 188 eligible patients for baseline analysis (**Table 1**) based on inclusion and exclusion criteria. The age cutoff for the patients was 58 years (range: 25-81 years), with 76.6% of the patients being 58 years or younger. The most common histological type was BC of no special type, observed in 177 patients (94.1%). Grade 2 was the predominant tumor pathological grade, seen in 132 patients (70.2%). The distribution of invasive BC stages was as follows: stage I, 27.7%; stage II, 43.6%; and stage III, 28.7%. Estrogen receptor (ER) positivity was identified in 64.9% of the patients, and progesterone receptor (PR) positivity was found in 57.4%, while 22.9% were positive for human epidermal growth factor receptor-2 (HER-2). Hormone receptor positive (HR+) means estrogen receptor positive or progesterone receptor positive or both. Hormone receptor negative (HR-) means estrogen receptor and progesterone receptor negative. In terms of intrinsic subtypes, HR+/HER2- was the most common, comprising 54.3% of the patients, followed by HR-/HER2- (22.9%), HR+/HER2+ (13.3%), and HR-/HER2+ (9.6%). A majority of patients (81.4%) had a Ki67 level greater than 20%. Regarding treatment, most patients (93.6%) underwent modified radical mastectomy, while 6.4% had partial mastectomy. Additionally, 96.8% of the patients received chemotherapy, 22.3% received targeted therapies, 62.8% received endocrine therapies, and 53.7% underwent radiotherapy. Hypertension and diabetes were present at the time of diagnosis in 18.1% and 6.4% of the patients, respectively. The median follow-up duration was 128 months (range: 27-188 months). During the follow-up period, 54 patients (28.7%) experienced disease recurrence, and 28 patients (14.9%) died.

**Table 1:** Baseline characteristics of patients of the whole study population.

Characteristic	All Patients (n=188)	Low SFI (n=131)	High SFI (n=57)	P value
Age (%)	50.06 (10.49)	48.03 (9.53)	54.72 (11.18)	
<=58	144 (76.6)	105 (80.2)	39 (68.4)	0.081
>58	44 (23.4)	26 (19.8)	18 (31.6)	
BMI (%)	22.90 (3.19)	21.97 (2.97)	25.03 (2.63)	
<23	108 (57.4)	96 (73.3)	12 (21.1)	**<0.001**
>=23	80 (42.6)	35 (26.7)	45 (78.9)	
BSA (mean (SD))	1.61 (0.12)	1.59 (0.11)	1.67 (0.12)	**<0.001**
NRS2002 (%)				
0	152 (80.9)	109 (83.2)	43 (75.4)	0.356
1	26 (13.8)	15 (11.5)	11 (19.3)	
2, 3	10 (5.3)	7 (5.3)	3 (5.3)	
Pathology (%)				
No Special Type	177 (94.1)	124 (94.7)	53 (93.0)	0.911
Special Type	11 (5.9)	7 (5.3)	4 (7.0)	
Tumor grade (%)				
1	12 (6.4)	10 (7.6)	2 (3.5)	0.330
2	132 (70.2)	88 (67.2)	44 (77.2)	
3	44 (23.4)	33 (25.2)	11 (19.3)	
T (%)				
T1	85 (45.2)	62 (47.3)	23 (40.4)	0.377
T2-3	103 (54.8)	69 (52.7)	34 (59.6)	
N (%)				
N0	94 (50.0)	71 (54.2)	23 (40.4)	**0.049**
N1	41 (21.8)	30 (22.9)	11 (19.3)	
N2-3	53 (28.2)	30 (22.9)	23 (40.4)	
Stage (%)				
1	52 (27.7)	42 (32.1)	10 (17.5)	**0.031**
2	82 (43.6)	58 (44.3)	24 (42.1)	
3	54 (28.7)	31 (23.7)	23 (40.4)	
ER (%)				
Negative	66 (35.1)	47 (35.9)	19 (33.3)	0.737
Positive	122 (64.9)	84 (64.1)	38 (66.7)	
PR (%)				
Negative	80 (42.6)	55 (42.0)	25 (43.9)	0.811
Positive	108 (57.4)	76 (58.0)	32 (56.1)	
HER2 (%)				
Negative	145 (77.1)	98 (74.8)	47 (82.5)	0.251
Positive	43 (22.9)	33 (25.2)	10 (17.5)	
Intrinsic subtype (%)				
HR-/HER2-	43 (22.9)	26 (19.8)	17 (29.8)	0.061
HR-/HER2+	18 (9.6)	17 (13.0)	1 (1.8)	
HR+/HER2+	25 (13.3)	16 (12.2)	9 (15.8)	
HR+/HER2-	102 (54.3)	72 (55.0)	30 (52.6)	
Ki67 (%)				
<=20	35 (18.6)	28 (21.4)	7 (12.3)	0.141
>20	153 (81.4)	103 (78.6)	50 (87.7)	
Operation (%)				
MRM	176 (93.6)	122 (93.1)	54 (94.7)	0.928
PM	12 (6.4)	9 (6.9)	3 (5.3)	
Chemotherapy (%)				
Not done	6 (3.2)	2 (1.5)	4 (7.0)	0.129
Done	182 (96.8)	129 (98.5)	53 (93.0)	
Targeted therapy (%)				
Not done	146 (77.7)	98 (74.8)	48 (84.2)	0.155
Done	42 (22.3)	33 (25.2)	9 (15.8)	
Endocrine therapy (%)				
Not done	70 (37.2)	47 (35.9)	23 (40.4)	0.560
Done	118 (62.8)	84 (64.1)	34 (59.6)	
Radiotherapy (%)				
Not done	87 (46.3)	65 (49.6)	22 (38.6)	0.164
Done	101 (53.7)	66 (50.4)	35 (61.4)	
Hypertension (%)				
NO	154 (81.9)	115 (87.8)	39 (68.4)	**0.002**
YES	34 (18.1)	16 (12.2)	18 (31.6)	
Diabetes (%)				
NO	176 (93.6)	123 (93.9)	53 (93.0)	0.757
YES	12 (6.4)	8 (6.1)	4 (7.0)	
Hemoglobin (mean (SD))	126.75 (15.60)	126.75 (16.41)	126.75 (13.68)	0.998
Total Protein (mean (SD))	71.10 (6.66)	71.01 (7.02)	71.32 (5.78)	0.774
Albumin (mean (SD))	39.84 (4.11)	40.02 (4.31)	39.42 (3.59)	0.357
T11SMI (mean (SD))	28.52 (4.06)	27.63 (3.93)	30.56 (3.61)	**<0.001**
T11VFI (mean (SD))	16.80 (11.71)	12.66 (7.94)	26.31 (13.40)	**<0.001**
T11SFI (mean (SD))	42.09 (15.09)	34.05 (9.08)	60.57 (8.40)	**<0.001**
T11IMFI (mean (SD))	3.10 (2.28)	2.41 (1.58)	4.70 (2.80)	**<0.001**

BMI, body mass index; BSA, body surface area; SD, standard deviation; NRS2002, nutritional risk screening 2002; T, primary tumor; N, nodal stage; Stage, cancer stage; ER, estrogen receptor; PR, progesterone receptor; HR, hormone receptor; HER2, human epidermal growth factor 2; MRM, modified radical mastectomy; PM, partial mastectomy; T11, the 11th thoracic vertebra level; SMI, skeletal muscle index; VFI, visceral fat index; SFI, subcutaneous fat index; IMFI, intermuscular fat index. The sum of the percentages is 100% ± 0.1% due to the mathematical characteristics of rounding. Bold values indicate statistical significance.

According to the optimal cutoff value for the SFI of 49.31 cm^2^/m^2^, patients were divided into two groups: the low SFI group (n = 131) and the high SFI group (n = 57). Several significant differences were observed between the two groups. First, BMI analysis revealed that 73.3% of patients in the low SFI group had a BMI below 23, whereas only 21.1% of those in the high SFI group had a BMI below 23 (*P* < 0.001). Conversely, 78.9% of patients in the high SFI group had a BMI of 23 or higher, compared to 26.7% in the low SFI group. Additionally, the mean BSA was significantly different between the groups, with the low SFI group having a mean BSA of 1.59 (SD = 0.11), compared to 1.67 (SD = 0.12) in the high SFI group (*P* < 0.001). Lymph nodal stage (N) also showed differences, with 54.2% of patients in the low SFI group at N0, compared to 40.4% in the high SFI group (*P* = 0.049). For BC stage, 32.1% of patients in the low SFI group were classified as stage I, compared to 17.5% in the high SFI group (*P* = 0.031). Hypertension was present in 12.2% of patients in the low SFI group, whereas 31.6% of those in the high SFI group had hypertension (*P* = 0.002), indicating a significantly higher prevalence of hypertension in the high SFI group. Additionally, SFI was positively correlated with SMI, VFI and IMFI (all *P* < 0.001). These baseline characteristics indicate clinically significant differences between the low and high SFI groups, which may influence patient outcomes.

### 3.2 Survival outcomes before propensity score matching

**Figure 4:**
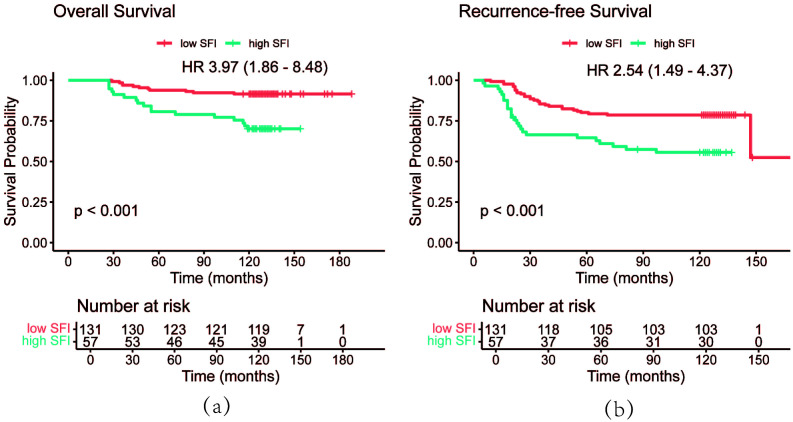
OS curves **(a)** and RFS curves **(b)** of patients by SFI. OS, overall survival; RFS, recurrence-free survival; SFI, subcutaneous fat index.

Using the optimal cutoff value for SFI, the data revealed that patients with an SFI below this threshold had significantly better prognoses than those with higher SFI. The 3-year, 5-year, and 10-year OS rates in the low SFI group were 96.9%, 93.9%, and 91.6%, respectively, compared to 91.2%, 80.7%, and 70.2% in the high SFI group. Similarly, the 3-year, 5-year, and 10-year RFS rates were 85.5%, 80.2%, and 78.6% in the low SFI group, compared to 66.4%, 64.6%, and 55.6% in the high SFI group. Kaplan-Meier survival analysis showed that patients in the low SFI group had significantly longer OS (*P* < 0.001) and RFS (*P* < 0.001) than those in the high SFI group (**Figure 4**).

Univariate Cox regression analysis identified five variables significantly affecting OS: age at diagnosis (*P* = 0.010), BMI (*P* = 0.016), stage (*P* = 0.001), radiotherapy (*P* = 0.020), and SFI (*P* < 0.001). Additionally, age at diagnosis (*P* = 0.033), BMI (*P* = 0.028), stage (*P* < 0.001), radiotherapy (*P* < 0.001), and SFI (*P* < 0.001) were important factors influencing RFS (**Table 2**). Multivariate Cox regression analysis was performed on variables with significant differences from the univariate analysis to eliminate confounding factors and identify independent predictors of RFS and OS (**Table 2**). The results indicated that age (HR 2.57, 95% CI 1.16-5.70, *P* = 0.020), stage (HR 4.22, 95% CI 1.01-17.71, *P* = 0.049), and SFI (HR 2.50, 95% CI 1.07-5.83, *P* = 0.034) were independent predictors of OS. Specifically, patients older than 58 years, those with stage III cancer, and those with high SFI were strongly associated with poor OS. Similarly, age (HR 1.85, 95% CI 1.02-3.34, *P* = 0.042), radiotherapy (HR 2.02, 95% CI 1.00-4.07, *P* = 0.048), and SFI (HR 2.04, 95% CI 1.10-3.78, *P* = 0.024) were independent factors affecting RFS. Specifically, patients over 58 years old and those with high SFI had poorer RFS. Additionally, patients who received radiotherapy had a higher risk of recurrence. The multivariate Cox proportional hazards analysis showed that high SFI is a significant predictor of poor RFS and OS in patients.

**Table 2:** Univariate and multivariate analyses on OS and on RFS.

Characteristic	Overall survival				Recurrence-free survival			
	Univariate Analyses		Multivariable analysis		Univariate Analyses		Multivariable analysis	
	HR (95% CI)	P value	HR (95% CI)	P value	HR (95% CI)	P value	HR (95% CI)	P value
Age								
<=58	1.00		1.00		1.00		1.00	
>58	2.66 (1.26-5.63)	**0.010**	2.57 (1.16-5.70)	**0.020**	1.86 (1.05-3.28)	**0.033**	1.85 (1.02-3.34)	**0.042**
BMI								
<23	1.00		1.00		1.00		1.00	
>=23	2.58 (1.19-5.58)	**0.016**	1.47 (0.62-3.46)	0.379	1.83 (1.07-3.15)	**0.028**	1.16 (0.62-2.17)	0.639
BSA	4.73 (0.21-104.69)	0.325			8.64 (0.95-78.22)	0.055		
NRS2002								
0	1.00				1.00			
1	1.38 (0.52-3.63)	0.519			0.70 (0.30-1.65)	0.419		
2, 3	0.67 (0.09-4.93)	0.690			0.61 (0.15-2.52)	0.498		
Pathology								
No Special Type	1.00				1.00			
Special Type	1.22 (0.29-5.13)	0.788			0.56 (0.14-2.31)	0.426		
Tumor grade								
1	1.00				1.00			
2	1.86 (0.25-13.89)	0.543			1.94 (0.47-8.02)	0.360		
3	1.96 (0.24-15.93)	0.529			1.32 (0.29-6.02)	0.721		
Stage								
1	1.00		1.00		1.00		1.00	
2	1.28 (0.32-5.14)	0.723	0.74 (0.17-3.28)	0.694	1.84 (0.81-4.14)	0.144	1.14 (0.47-2.76)	0.767
3	7.37 (2.18-24.93)	**0.001**	4.22 (1.01-17.71)	**0.049**	3.90 (1.76-8.65)	**<0.001**	2.02 (0.81-5.03)	0.130
ER								
Negative	1.00				1.00			
Positive	0.92 (0.43-2.00)	0.836			1.03 (0.59-1.82)	0.911		
PR								
Negative	1.00				1.00			
Positive	0.60 (0.29-1.27)	0.184			0.85 (0.49-1.46)	0.551		
HER2								
Negative	1.00				1.00			
Positive	1.41 (0.62-3.20)	0.414			1.00 (0.52-1.90)	0.991		
Intrinsic subtype								
HR-/HER2-	1.00				1.00			
HR-/HER2+	2.08 (0.56-7.73)	0.276			1.41 (0.51-3.87)	0.510		
HR+/HER2+	1.36 (0.36-5.05)	0.649			0.99 (0.36-2.74)	0.991		
HR+/HER2-	1.23 (0.45-3.40)	0.683			1.24 (0.61-2.52)	0.560		
Ki67								
<=20	1.00				1.00			
>20	1.07 (0.41-2.80)	0.898			2.03 (0.87-4.75)	0.102		
Chemotherapy								
Not done	1.00				1.00			
Done	0.41 (0.10-1.73)	0.226			0.49 (0.15-1.58)	0.234		
Targeted therapy								
Not done	1.00				1.00			
Done	1.03 (0.44-2.43)	0.941			0.90 (0.46-1.75)	0.763		
Targeted therapy								
Not done	1.00				1.00			
Done	0.87 (0.41-1.86)	0.723			1.12 (0.63-1.97)	0.700		
Radiotherapy								
Not done	1.00		1.00		1.00		1.00	
Done	2.77 (1.18-6.52)	**0.020**	1.29 (0.46-3.65)	0.633	2.81 (1.53-5.16)	**<0.001**	2.02 (1.00-4.07)	**0.048**
Hypertension								
NO	1.00				1.00			
YES	1.92 (0.84-4.35)	0.120			1.38 (0.73-2.63)	0.325		
Diabetes								
NO	1.00				1.00			
YES	2.75 (0.95-7.92)	0.061			1.23 (0.44-3.42)	0.687		
Hemoglobin	1.01 (0.99-1.03)	0.514			0.99 (0.98-1.01)	0.544		
Total Protein	1.01 (0.95-1.07)	0.740			1.00 (0.96-1.04)	0.926		
Albumin	0.97 (0.89-1.06)	0.543			0.98 (0.92-1.04)	0.487		
T11SFI								
Low	1.00		1.00		1.00		1.00	
High	3.97 (1.86-8.48)	**<0.001**	2.50 (1.07-5.83)	**0.034**	2.54 (1.49-4.37)	**<0.001**	2.04 (1.10-3.78)	**0.024**

BMI, body mass index; BSA, body surface area; SD, standard deviation; NRS2002, nutritional risk screening 2002; Stage, cancer stage; ER, estrogen receptor; PR, progesterone receptor; HR, hormone receptor; HER2, human epidermal growth factor 2; T11, the 11th thoracic vertebra level; SFI, subcutaneous fat index. Bold values indicate statistical significance.

### 3.3 Survival outcomes after propensity score matching

**Figure 5:**
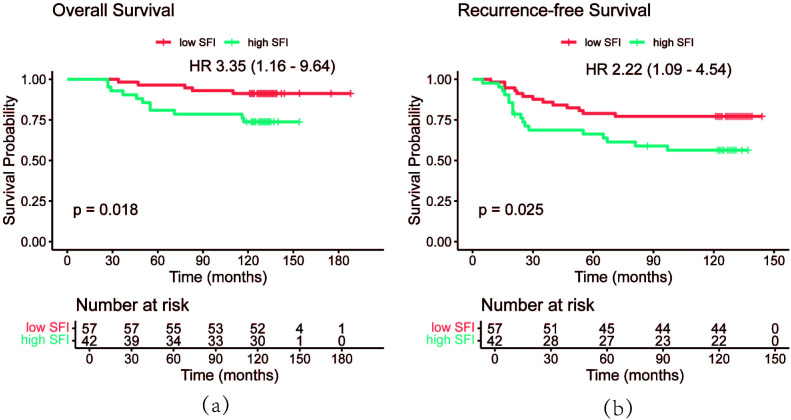
OS curves **(a)** and RFS **(b)** curves of patients after PSM by SFI. OS, overall survival; RFS, recurrence-free survival; PSM, propensity score matching; SFI, subcutaneous fat index.

PSM analysis was performed based on BMI, BSA, disease stage, and hypertension status to minimize random and systematic errors (**Table 3**). After PSM, the 3-year, 5-year, and 10-year OS rates in the high SFI group were 92.9%, 81.0%, and 73.8%, respectively. In contrast, the low SFI group had 3-year, 5-year, and 10-year OS rates of 98.2%, 96.5%, and 91.2%, respectively. Similarly, the 3-year, 5-year, and 10-year RFS rates in the low SFI group were 86.0%, 78.9%, and 77.2%, while the high SFI group had corresponding RFS rates of 68.8%, 66.3%, and 56.4%. Kaplan-Meier survival curve analysis revealed significant differences in both RFS and OS between the two groups (*P* = 0.025 and *P* = 0.018, respectively, **Figure 5**). These findings were consistent with the results obtained before PSM analysis. Overall, patients with a higher SFI had poorer 3-, 5-, and 10-year OS and RFS, indicating that SFI may serve as a potential predictor of poor prognosis in postoperative BC patients.

**Table 3:** Baseline characteristics of patients in the propensity score matching cohort.

Characteristic	All Patients (n=99)	Low SFI (n=57)	High SFI (n=42)	P value
Age (%)				
<=58	73 (73.7)	45 (78.9)	28 (66.7)	0.170
>58	26 (26.3)	12 (21.1)	14 (33.3)	
BMI (%)				
<23	33 (33.3)	22 (38.6)	11 (26.2)	0.196
>=23	66 (66.7)	35 (61.4)	31 (73.8)	
BSA (mean (SD))	1.65 (0.11)	1.65 (0.11)	1.66 (0.12)	0.719
NRS2002 (%)				
0	80 (80.8)	49 (86.0)	31 (73.8)	0.289
1	15 (15.2)	6 (10.5)	9 (21.4)	
2, 3	4 (4.0)	2 (3.5)	2 (4.8)	
Pathology (%)				
No Special Type	91 (91.9)	53 (93.0)	38 (90.5)	0.937
Special Type	8 (8.1)	4 (7.0)	4 (9.5)	
Tumor grade (%)				
1	3 (3.0)	2 (3.5)	1 (2.4)	0.675
2	68 (68.7)	37 (64.9)	31 (73.8)	
3	28 (28.3)	18 (31.6)	10 (23.8)	
T (%)				
T1	41 (41.4)	23 (40.4)	18 (42.9)	0.802
T2-3	58 (58.6)	34 (59.6)	24 (57.1)	
N (%)				
N0	44 (44.4)	24 (42.1)	20 (47.6)	0.641
N1	24 (24.2)	13 (22.8)	11 (26.2)	
N2-3	31 (31.3)	20 (35.1)	11 (26.2)	
Stage (%)				
1	24 (24.2)	14 (24.6)	10 (23.8)	0.454
2	43 (43.4)	22 (38.6)	21 (50.0)	
3	32 (32.3)	21 (36.8)	11 (26.2)	
ER (%)				
Negative	40 (40.4)	23 (40.4)	17 (40.5)	0.990
Positive	59 (59.6)	34 (59.6)	25 (59.5)	
PR (%)				
Negative	48 (48.5)	24 (42.1)	24 (57.1)	0.139
Positive	51 (51.5)	33 (57.9)	18 (42.9)	
HER2 (%)				
Negative	82 (82.8)	44 (77.2)	38 (90.5)	0.083
Positive	17 (17.2)	13 (22.8)	4 (9.5)	
Intrinsic subtype (%)				
HR-/HER2-	31 (31.3)	14 (24.6)	17 (40.5)	0.082
HR-/HER2+	6 (6.1)	6 (10.5)	0 (0.0)	
HR+/HER2+	11 (11.1)	7 (12.3)	4 (9.5)	
HR+/HER2-	51 (51.5)	30 (52.6)	21 (50.0)	
Ki67 (%)				
<=20	13 (13.1)	7 (12.3)	6 (14.3)	0.770
>20	86 (86.9)	50 (87.7)	36 (85.7)	
Operation (%)				
MRM	93 (93.9)	54 (94.7)	39 (92.9)	0.696
PM	6 (6.1)	3 (5.3)	3 (7.1)	
Chemotherapy (%)				
Not done	3 (3.0)	0 (0.0)	3 (7.1)	0.145
Done	96 (97.0)	57 (100.0)	39 (92.9)	
Targeted therapy (%)				
Not done	82 (82.8)	44 (77.2)	38 (90.5)	0.083
Done	17 (17.2)	13 (22.8)	4 (9.5)	
Endocrine therapy (%)				
Not done	42 (42.4)	21 (36.8)	21 (50.0)	0.190
Done	57 (57.6)	36 (63.2)	21 (50.0)	
Radiotherapy (%)				
Not done	41 (41.4)	24 (42.1)	17 (40.5)	0.871
Done	58 (58.6)	33 (57.9)	25 (59.5)	
Hypertension (%)				
NO	77 (77.8)	47 (82.5)	30 (71.4)	0.192
YES	22 (22.2)	10 (17.5)	12 (28.6)	
Diabetes (%)				
NO	91 (91.9)	52 (91.2)	39 (92.9)	0.999
YES	8 (8.1)	5 (8.8)	3 (7.1)	
Hemoglobin (mean (SD))	126.56 (12.38)	127.11 (10.84)	125.81 (14.31)	0.609
Total Protein (mean (SD))	71.71 (6.00)	71.67 (6.54)	71.77 (5.27)	0.938
Albumin (mean (SD))	39.88 (4.05)	39.85 (4.36)	39.92 (3.63)	0.925
T11SMI (mean (SD))	29.67 (3.95)	29.11 (4.17)	30.44 (3.53)	0.097
T11VFI (mean (SD))	19.25 (10.51)	15.52 (8.47)	24.32 (10.97)	**<0.001**
T11SFI (mean (SD))	47.14 (13.77)	37.41 (7.85)	60.33 (7.68)	**<0.001**
T11IMFI (mean (SD))	3.40 (2.56)	2.42 (1.79)	4.73 (2.86)	**<0.001**

BMI, body mass index; BSA, body surface area; SD, standard deviation; NRS2002, nutritional risk screening 2002; T, primary tumor; N, nodal stage; Stage, cancer stage; ER, estrogen receptor; PR, progesterone receptor; HR, hormone receptor; HER2, human epidermal growth factor 2; MRM, modified radical mastectomy; PM, partial mastectomy; T11, the 11th thoracic vertebra level; SMI, skeletal muscle index; VFI, visceral fat index; SFI, subcutaneous fat index; IMFI, intermuscular fat index. Bold values indicate statistical significance.

### 3.4 Subgroup analysis for clinical impact of SFI

Subgroup analyses were conducted based on BMI at diagnosis, hormone receptor status, and diabetes mellitus to examine the correlation between T11SFI and prognosis in each subgroup (**Table 4**). The results showed that the impact of SFI on BC prognosis differed across BMI levels. In patients with a BMI < 23, a high SFI significantly increased the risk of recurrence (HR 3.59, 95% CI 1.42-9.05, *P* = 0.007). However, this association was not significant in patients with a BMI ≥ 23 (HR 1.72, 95% CI 0.80-3.70, *P* = 0.166). Although a trend toward increased mortality risk was observed, the effect of higher SFI on OS did not reach statistical significance in either BMI subgroup (BMI < 23: HR 3.68, 95% CI 0.95-14.25, *P* = 0.059; BMI ≥ 23: HR 3.04, 95% CI 1.00-9.24, *P* = 0.050). In the subgroup analysis based on hormone receptor status, a higher SFI was significantly associated with an increased risk of death (HR 3.33, 95% CI 1.34-8.29, *P* = 0.010) and recurrence (HR 2.02, 95% CI 1.06-3.88, *P* = 0.034) in hormone receptor positive patients. Similarly, in hormone receptor negative patients, higher SFI was linked to an elevated risk of death (HR 5.65, 95% CI 1.41-22.62, *P* = 0.014) and recurrence (HR 4.03, 95% CI 1.50-10.87, *P* = 0.006). For patients without diabetes, higher SFI was significantly associated with an increased risk of recurrence (HR 2.52, 95% CI 1.44-4.42, *P* = 0.001) and death (HR 4.27, 95% CI 1.87-9.77, *P* < 0.001). In contrast, for patients with diabetes, higher SFI was not significantly associated with recurrence risk (HR 2.94, 95% CI 0.41-21.24, *P* = 0.285) or mortality risk (HR 2.57, 95% CI 0.36-18.42, *P* = 0.348).

**Table 4:** Prognostic impact of T11SFI according to BMI, Hormone receptor and diabetes group.

Characteristic	T11SFI	Overall survival		Recurrence-free survival	
		HR (95% CI)	P value	HR (95% CI)	P value
BMI					
<23	Low	1.00		1.00	
	High	3.68(0.95-14.25)	0.059	3.59(1.42-9.05)	**0.007**
>=23	Low	1.00		1.00	
	High	3.04(1.00-9.24)	0.050	1.72(0.80-3.70)	0.166
Hormone receptor					
Negative	Low	1.00		1.00	
	High	5.65(1.41-22.62)	**0.014**	4.03(1.50-10.87)	**0.006**
Positive	Low	1.00		1.00	
	High	3.33(1.34-8.29)	**0.010**	2.02(1.06-3.88)	**0.034**
Diabetes					
NO	Low	1.00		1.00	
	High	4.27(1.87-9.77)	**<0.001**	2.52(1.44-4.42)	**0.001**
YES	Low	1.00		1.00	
	High	2.57(0.36-18.42)	0.348	2.94(0.41-21.24)	0.285

Bold values indicate statistical significance.

## 4 Discussion

To date, no systematic studies have explored the relationship between the SFI at the T11 level and overall prognosis in BC patients. The SFI, calculated as the area of subcutaneous fat divided by the square of height, allows for the assessment of subcutaneous obesity while accounting for height. While prior studies have examined SFA as a prognostic factor for certain cancers, the role of SFI has not been thoroughly investigated. Our study highlights the significance of SFI as a prognostic factor in BC and offers a new perspective for individualized diagnosis and treatment. Using propensity score matching, we systematically assessed the influence of subcutaneous fat tissue at the T11 plane on BC prognosis. The results demonstrated that a higher SFI at the T11 level was significantly associated with poorer OS and RFS in BC patients. Specifically, patients with a higher SFI had worse prognoses. This was consistently shown through Kaplan-Meier survival curves, multivariate Cox regression analysis, and PSM analysis, all of which indicated that excessive subcutaneous fat may adversely affect the OS and RFS of patients with stage I-III BC after surgery.

So far, only a limited number of studies have explored the relationship between subcutaneous fat tissue and cancer prognosis, with findings remaining contentious. Some studies suggest that lower levels of subcutaneous fat are associated with higher mortality rates and poorer survival outcomes. For instance, a retrospective cohort study of 1,473 patients with gastrointestinal and respiratory cancers, as well as 273 patients with metastatic renal cell carcinoma, found that a reduced SFI was linked to increased mortality and shorter survival (36). Similarly, Lopez et al. conducted a systematic review and meta-analysis to examine the impact of fat and muscle mass on survival outcomes in prostate cancer patients, revealing that a higher SFI was associated with improved OS. However, while their meta-analytic design strengthened the robustness of their findings, it also limited their ability to precisely control for patient characteristics due to the inherent diversity within the sample (37). In BC research, A retrospective study involving 517 BC patients further suggested that a low T11 SFI might predict bone metastasis in BC (2). Contrarily, our study found that patients with a high SFI had a poorer prognosis. A prospective cohort study by Bradshaw et al. supported this finding, demonstrating that an increased SFA might be linked to reduced survival rates in BC patients, emphasizing the role of fat distribution in BC prognosis (38). Furthermore, Cheng et al. identified a correlation between higher subcutaneous adipose tissue density (SATD) and an increased risk of overall mortality in BC patients, suggesting the potential predictive value of SATD for BC prognosis (39). The discrepancies observed in studies investigating high versus low SFI may be due to differences in study design, patient populations, and outcome measures. For example, certain studies, including that of Lopez et al. (37), employed systematic reviews or meta-analyses to synthesize data from multiple sources, enhancing generalizability. While this approach is beneficial, it also presents challenges in uniformly controlling for individual patient characteristics across different samples. Moreover, the types of cancers studied varied, with some studies focusing on specific cancers, which could affect the disease’s metabolic status and progression. For example, the unique pathological mechanisms of BC may significantly influence prognosis. Studies suggest that breast adipose tissue is more strongly correlated with trunk subcutaneous fat, whereas its independent association with visceral fat or cardiometabolic risk factors is weaker (40). Moreover, crown-like structures in breast adipose tissue (CLS-B), which are inflammatory markers formed by macrophages surrounding dead adipocytes, may create a favorable environment for tumor growth by promoting inflammation and estrogen production (41). Regarding study populations, most of the aforementioned studies (2, 36, 37) focused on patients with advanced cancer, who are more likely to experience increased fat consumption, which may result in poorer outcomes in those with low subcutaneous fat. Cancer patients often undergo significant weight changes and fat redistribution in advanced stages, which may negatively affect prognosis (42–44). In terms of outcome measures, some studies focused on fat density (39), while others examined fat volume or area, which may lead to differing interpretations of how SFI impacts cancer prognosis.

The underlying the association between a high SFI and poor prognosis in BC may involve multiple factors, including inflammatory responses and adipokine dysregulation. Excessive subcutaneous fat leads to the expansion of subcutaneous adipocytes, which, at a certain threshold, causes adipocyte damage that triggers chronic inflammation. During this process, immune cells, such as macrophages, accumulate around necrotic or damaged adipocytes, forming crown-like structures (CLS). These structures release pro-inflammatory factors, including interleukins (IL-6, IL-8), monocyte chemotactic protein (MCP-1), and tumor necrosis factor alpha (TNFα), which promote a pro-inflammatory environment in the breast tumor microenvironment, thereby facilitating the development of BC (45). The aggregation of macrophages, T cells, and B cells in these inflammatory CLS within breast adipose tissue correlates with the severity of obesity-induced insulin resistance (OIR), a metabolic disorder that increases mortality from various diseases, including cancer (46). In addition, the rapid expansion of subcutaneous fat often leads to localized hypoxia and the activation of hypoxia-inducible factor (HIF-1), which hinders preadipocyte differentiation and triggers adipose tissue fibrosis. This hypoxic and fibrotic environment not only attracts more immune cells but also generates pro-inflammatory signals that contribute to the growth and migration of BC cells (47). Subcutaneous fat, classified as white adipose tissue, plays important endocrine roles and secretes large amounts of hormones and other factors, such as leptin, collectively referred to as adipokines. Plasma leptin levels increase proportionally with total adipose tissue mass (48). High subcutaneous adiposity leads to elevated leptin levels (hyperleptinemia), a condition that promotes the secretion of inflammatory factors and further activates immune cells, thus affecting the tumor immune microenvironment and supporting cancer cell growth and migration. Key signaling pathways involved include Janus kinase 2-signal transducer and activator of transcription 3 (JAK2-STAT3), mitogen-activated protein kinase (MAPK), and phosphatidylinositol 3-kinase-protein kinase B (PI3K-AKT) (49). In conclusion, subcutaneous fat contributes directly or indirectly to the development and progression of BC through various mechanisms, including inflammatory responses, leptin signaling, tumor microenvironment remodeling, and hypoxia induction.

In this subgroup analysis, we found that variations in BMI, hormone receptor status, and diabetes influence the impact of subcutaneous fat on BC prognosis. In patients with a BMI < 23, higher subcutaneous fat was significantly associated with an increased risk of recurrence, suggesting that subcutaneous fat has a more pronounced prognostic impact by affecting metabolism and inflammatory status in individuals with lower BMI. This aligns with the findings of Picon-Ruiz et al. (50), who observed that obesity can increase BC aggressiveness and metastasis by promoting inflammation and metabolic disturbances. In contrast, the correlation between subcutaneous fat and recurrence risk was not significant in patients with a BMI ≥ 23. This may be because other metabolic factors, such as insulin resistance and lipid metabolism disorders, as well as concurrent chronic conditions like type 2 diabetes, play a more decisive role in the prognosis of patients with higher BMI (51), thereby overshadowing the independent effect of subcutaneous fat. These results suggest that BMI acts as a key modifier of the prognostic influence of subcutaneous fat on BC, closely tied to the individual’s metabolic status. This highlights the importance of considering individualized metabolic profiles and body fat distribution when formulating treatment and follow-up plans for BC patients, particularly in those with low BMI, where subcutaneous fat plays a significant role.

Furthermore, our study found that higher subcutaneous fat significantly increased the risk of recurrence and death in patients with hormone receptor negative BC. This may be due to the fact that obesity-induced chronic inflammation and metabolic dysregulation are more likely to promote tumor invasiveness and metastasis in hormone receptor negative cases. Several studies have shown that premenopausal obese women are at higher risk of recurrence and death in hormone receptor negative BC (52, 53). In addition, hormone receptor negative BC is typically more aggressive and prone to metastasis (54), which may amplify the effects of obesity-related inflammation and metabolic disorders in this type of tumor. Conversely, in hormone receptor positive BC, while a high SFI remains a prognostic disadvantage, its negative impact may be mitigated by endocrine therapy. Long-term use of treatments like tamoxifen has been shown to effectively control hormone receptor positive BC and reduce the risk of recurrence and death (55), potentially lessening the prognostic impact of high SFI in these patients. Subcutaneous fat is a significant source of estrogen. An increase in subcutaneous fat tissue induces chronic inflammation and the secretion of pro-inflammatory factors, which in turn stimulate aromatase expression and activity, leading to elevated estradiol levels (56). Furthermore, *in vitro* experiments have found that leptin secreted by subcutaneous adipose tissue regulates estrogen synthesis by upregulating aromatase gene expression and activity in MCF-7 cells, thereby enhancing estrogen synthesis (57). In HR-positive BC, subcutaneous fat may elevate estrogen levels through locally increased aromatase expression, which subsequently promotes tumor growth. Conversely, in HR-negative BC, the release of pro-inflammatory factors (e.g., IL-6 and TNF-α) from subcutaneous fat in an obese environment significantly enhances the invasive potential of BC cells and increases chemotherapy resistance (58). In HR-negative BC patients, the pro-inflammatory environment induced by subcutaneous fat predisposes tumors to adapt and evade immune surveillance, thereby complicating disease management. As a result, interventions targeting obesity-associated subcutaneous fat and metabolic pathways (e.g., modulation of adipokines or inflammatory responses) have emerged as potential therapeutic strategies (49), particularly for treating HR-negative BC patients.

In patients without diabetes, higher subcutaneous fat was significantly associated with increased risk of recurrence and death, suggesting that the impact of subcutaneous fat on prognosis is more pronounced in individuals with relatively normal metabolic status. Goodwin et al. found that high fasting insulin levels were linked to higher distant recurrence and mortality in a cohort of 512 women without diagnosed diabetes but with early-stage BC (59). This suggests that in metabolically normal patients, adipose tissue may influence prognosis via the insulin signaling pathway. However, in patients with diabetes, this effect may be obscured by the complex pathological processes of the disease. Lipscombe et al. (60) showed that diabetes was associated with a nearly 40% increase in mortality within the first five years after BC surgery, a rate similar to that of diabetic women without BC. The intricate metabolic imbalances and complications present in diabetic patients may overshadow the effect of subcutaneous fat on BC prognosis. Subcutaneous fat tissue has a limited storage capacity, and excessive caloric intake beyond this threshold leads to fat accumulation in ectopic tissues. For instance, excessive lipid buildup in the pancreas can result in β-cell dysfunction, causing localized inflammation and insulin resistance, which in turn promotes the development of diabetes mellitus (61). Obese individuals also tend to have higher insulin levels, which cause BC cells to produce more insulin-like growth factor 1 (IGF-1). Elevated IGF-1 stimulates the RAS/RAF/MAPK and PI3K/AKT/mTOR signaling pathways, potentially contributing to resistance to endocrine therapy (62). Consequently, clinicians should remain vigilant for new-onset diabetes in BC patients, as this may indicate a poorer prognosis.

This study identified subcutaneous fat at the T11 plane as an important indicator of BC prognosis. Although the effects of exercise, diet, and nutritional support on subcutaneous fat were not directly assessed, our findings provide a foundation for future research to explore how these lifestyle factors, by altering subcutaneous fat, may influence BC outcomes. This could help address the limitations of this study and offer a more comprehensive approach to BC management. Current intervention studies have shown that reducing fat mass improves metabolic and inflammatory markers, sex hormone levels, and breast density, thereby lowering the risk of BC (63–65). Harvie et al. (66) highlighted that adopting a healthy diet and lifestyle can significantly reduce the incidence of BC. Physical activity has also been shown to decrease BC incidence and impact recurrence or survival post-diagnosis (67). Exercise promotes a healthier distribution of body fat, increases bone mass (68), reduces inflammatory markers, and improves cardiovascular health (69), all of which contribute to improved patient outcomes. Additionally, preoperative and postoperative nutritional support can help regulate body fat distribution and improve treatment outcomes. Guidelines published by the ESPEN emphasize the crucial role of nutritional support in cancer treatment, noting that it not only improves nutritional status but also improves treatment tolerance and efficacy (70). These nutritional interventions, such as high-protein diets and supplementation with key nutrients, help patients maintain healthy fat distribution and metabolism during surgery and treatment. Chen et al. (71) further stressed that preoperative nutritional optimization can improve surgical tolerance and speed up recovery. Although imaging data suggest that managing subcutaneous fat through a healthy lifestyle may improve BC prognosis, this hypothesis needs to be validated by empirical studies. Future research should focus on optimizing BC treatment by investigating the potential benefits of combining lifestyle interventions with imaging assessments to improve patient outcomes. For example, future studies could be designed as randomized controlled trials with at least 150-200 participants per group. Specific lifestyle interventions to be tested could include tailored exercise programs (e.g., 150 minutes of moderate-intensity aerobic exercise per week), personalized nutritional guidance post-surgery (e.g., enteral high-protein dietary regimens), dietary modifications (e.g., high-fiber, low-sugar plans incorporating healthy fats), and regular mental health counseling (e.g., weekly sessions with a psychiatrist focusing on emotional regulation and coping skills training). Expected outcomes will focus on evaluating the impact of these lifestyle interventions on subcutaneous fat levels, measured periodically through imaging techniques (e.g., CT or MRI), as well as monitoring prognostic indicators such as survival, recurrence, and quality of life in BC patients. It is anticipated that exercise, dietary modifications, nutritional support, and psychological interventions will contribute to reducing subcutaneous fat, thus providing a more comprehensive approach to BC management.

Regular chest CT follow-ups after BC surgery (e.g., every 3 months for 2 years, every 6 months for years 3-5, and annually thereafter) provide healthcare providers with an effective opportunity to monitor changes in a patient’s SFI, forming the foundation for a more comprehensive and personalized rehabilitation management approach. Based on this study’s findings, which identified subcutaneous fat in the T11 plane as a prognostic indicator for BC, healthcare teams can use follow-up data to track lateral changes in SFI and integrate them into follow-up programs as a dynamic prognostic indicator. On this basis, healthcare professionals can emphasize the importance of a healthy lifestyle during patient counseling and provide customized dietary and exercise recommendations tailored to the patient’s body fat distribution, particularly for early-stage patients and high-risk groups. In addition, healthcare professionals can incorporate nutritional support and mental health interventions into treatment planning, making timely adjustments in coordination with body fat monitoring results. Collaboration among interdisciplinary teams—including oncologists, dietitians, exercise therapists, and mental health specialists—ensures a more tailored, patient-centered approach that continuously refines treatment plans. A comprehensive management model that combines SFI monitoring with lifestyle modifications facilitates the early identification of recurrence risk, supports patients in optimizing body fat levels, and ultimately enhances BC management outcomes and overall quality of life.

Our study had several limitations. First, due to its retrospective design, a significant number of patients were excluded due to unavailable chest CT images, potentially introducing selection bias. Since the use of PSM may reduce sample size, we recommend expanding the initial sample size to improve statistical power before conducting the study. Second, due to resource constraints, we could only calculate the SFI using CT scans and were unable to assess subcutaneous fat thickness via MRI or other methods. Third, the pathological data were recorded several years ago, meaning some modern diagnostic techniques, such as immunohistochemical staining and FISH testing, were not available. Race, marital status, average annual household income, menopausal status, inflammation levels, and basal metabolic rate may influence the relationship between subcutaneous fat and BC prognosis; however, we were unable to collect this information. Additionally, there is no widely recognized cutoff for SFI. Unlike the established threshold for L3 sarcopenia, this study relied on optimal cutoff values based on the data set, which may affect the generalizability and comparability of the results.

Despite these limitations, this study has notable strengths. It highlights a significant negative association between the SFI at the T11 plane and BC prognosis. These findings provide new evidence that managing subcutaneous fat through lifestyle interventions may improve outcomes for BC patients. To address the study’s limitations, future research should utilize more comprehensive imaging techniques, such as MRI, to assess subcutaneous fat more accurately. Alternatively, skinfold thickness can be used to initially assess a patient’s subcutaneous fat. While both skinfold thickness and SFI are common methods for evaluating subcutaneous fat, they differ in measurement principles, accuracy, and applicability. Skinfold thickness measures the thickness of skin and subcutaneous tissue at specific sites using calipers. This method is simple and convenient for screening and physical exams but is sensitive to variations in measurement sites and skinfold grasping techniques (72). In contrast, imaging-based SFI provides more accurate data on subcutaneous fat distribution by using standardized cross-sections. This makes it less susceptible to human error or body size factors, making it more suitable for precise clinical management and research. While both methods may correlate in assessing total subcutaneous fat, differences in individual fat distribution, measurement consistency, and methodology can impact reliability. For example, skinfold thickness may underestimate body fat in patients with uneven fat distribution or obesity. Moreover, the high reproducibility and accuracy of imaging-based SFI offer significant advantages in disease prediction and patient follow-up. Overall, skinfold thickness is suitable for initial screening, while SFI is better for cases requiring detailed fat assessment. The combined use of both methods can offer complementary insights. Moreover, developing standardized cutoff values for SFI would improve the comparability and applicability of study results. A parallel study examining the impact of subcutaneous fat distribution on outcomes across different anticancer treatments could further validate and refine the qualitative findings of this research.

## 5 Conclusions

A higher SFI may be associated with decreased OS and RFS in patients with stage I-III BC. Prevention and treatment strategies targeting subcutaneous fat may help improve patient outcomes.

## Data Availability

The raw data supporting the conclusions of this article will be made available by the authors, without undue reservation.
